# Proton-sensing ion channels, GPCRs and calcium signaling regulated by them: implications for cancer

**DOI:** 10.3389/fcell.2024.1326231

**Published:** 2024-03-05

**Authors:** Renhui Ji, Li Chang, Caiyan An, Junjing Zhang

**Affiliations:** ^1^ Foundational and Translational Medical Research Center, Department of Allergy and General Surgery, Hohhot First Hospital, Hohhot, China; ^2^ Department of Pathophysiology, Basic Medicine College of Inner Mongolia Medical University, Hohhot, China

**Keywords:** Ca^2+^ signaling, proton-sensing GPCRs, ion channels, acidic tumor microenvironment, tumor progression

## Abstract

Extracellular acidification of tumors is common. Through proton-sensing ion channels or proton-sensing G protein-coupled receptors (GPCRs), tumor cells sense extracellular acidification to stimulate a variety of intracellular signaling pathways including the calcium signaling, which consequently exerts global impacts on tumor cells. Proton-sensing ion channels, and proton-sensing GPCRs have natural advantages as drug targets of anticancer therapy. However, they and the calcium signaling regulated by them attracted limited attention as potential targets of anticancer drugs. In the present review, we discuss the progress in studies on proton-sensing ion channels, and proton-sensing GPCRs, especially emphasizing the effects of calcium signaling activated by them on the characteristics of tumors, including proliferation, migration, invasion, metastasis, drug resistance, angiogenesis. In addition, we review the drugs targeting proton-sensing channels or GPCRs that are currently in clinical trials, as well as the relevant potential drugs for cancer treatments, and discuss their future prospects. The present review aims to elucidate the important role of proton-sensing ion channels, GPCRs and calcium signaling regulated by them in cancer initiation and development. This review will promote the development of drugs targeting proton-sensing channels or GPCRs for cancer treatments, effectively taking their unique advantage as anti-cancer drug targets.

## 1 Introduction

The extracellular acid‒base state (pH) of most normal tissues in the body remains relatively stable, usually between 7.3 and 7.4 (Boedtkjer and Pedersen, 2020). However, acidification is prevalent in the extracellular microenvironment of malignant tumor tissues, with pH values as low as 6.3. Pampus (1963) Malignant tumors are characterized by vigorous growth, hypoplasia of blood vessels, severe hypoxia of tissues, and that energy is predominantly supplied through anaerobic glycolysis in the tumors’ metabolism, eventually leading to the formation of extracellular acidic microenvironments of the tumors. The acidic tumor microenvironment, in turn, accelerates tumor progression in multiple ways. For example, some products excreted from the acidic microenvironment, such as cathepsin B and other proteolytic enzymes, cause degradation of the extracellular matrix and necrosis and apoptosis of surrounding normal cells. Increased matrix metalloproteinase (MMP) activity promotes tumor cell invasion and migration (Boedtkjer and Pedersen, 2020). The acidic microenvironment also inhibits immune cell proliferation and drives immune escape by inducing dedifferentiation through epigenetic regulation, as well as autocrine and paracrine changes in cell function. In addition, the acidic microenvironment can alter drug structure and charge, thereby reducing their uptake into tumor cells and affecting the delivery and efficacy of anticancer drugs as well as chemotherapy and radiotherapy (Wang J. X. et al., 2020; Worsley et al., 2022). There are some ion channels or G protein-coupled receptors (GPCRs) on the cell membrane that can sense the extracellular acidic microenvironment, namely, proton-sensing ion channels or proton-sensing GPCRs. They are activated in acidic environments and then activate multiple downstream intracellular signaling pathways, including calcium signaling, to affect cell functions. Ca^2+^ is the most abundant second messenger in the human body, and controls a variety of cellular functions, such as cell differentiation, migration, proliferation, autophagy, apoptosis and gene expression (Berridge et al., 2000; Wang Y. et al., 2020; Emrich et al., 2022). Meanwhile, Ca^2+^ signaling is also a key regulator of many cancers and plays an important role in the proliferation, migration and invasion of cancer cells (Panda et al., 2022).

Ion channels or GPCRs have natural advantages as drug targets. First, they are localized to the cell membrane, therefore, targeted drugs do not need to be designed to cross the plasma membrane and are consequently easy to develop. Arcangeli and Becchetti (2015) Second, the size, shape, amino acid composition, and location of the orthosteric binding sites of GPCRs on the outside of the cell make GPCRs well suited for binding by small molecule synthetic compounds. In addition, the dynamic changes in receptor conformation among GPCRs upon activation also make them ideal drug targets (Congreve et al., 2020). Therefore, GPCRs have become the most intensively studied drug target, and approximately 35% of drugs approved by the Food and Drug Administration (FDA) target GPCRs for various indications, such as cancer, pain, allergy, hypertension, and neuropsychiatric disorders (Sriram and Insel, 2018; Davenport et al., 2020; Usman et al., 2020). Proton-sensing ion channels and GPCRs also have the above mentioned general advantages as drug targets. Additionally, proton-sensing ion channels and GPCRs play an important role in regulating tumor characteristics in response to the acidic tumor microenvironment, and have unique advantages compared with other ion channels or GPCRs. However, no anticancer drugs targeting proton-sensing ion channels or proton-sensing GPCRs have been developed in the past decades, and very few anticancer drugs are currently being examined in ongoing clinical trials. Therefore, this article reviews the important role of proton-sensing ion channels or GPCRs and their activated intracellular signaling pathways, with a special emphasis on the calcium signaling pathway and its influence on tumor properties (proliferation, migration, drug resistance, angiogenesis, etc.) (Tables 1, 2). In addition, with the development of novel experimental approaches such as cryo-electron microscopy (cryo-EM) (Zhang et al., 2021; Yelshanskaya and Sobolevsky, 2022), bioluminescence resonance energy transfer (BRET), and NanoBiT, (Tao et al., 2023) etc., great achievements have been made recently, especially in pharmaceutical studies. For example, cryo-EM technology made it possible to resolve the complex structure of channels/GPCRs and their ligands small molecules and protein-binding partners, leading to a large number of discoveries of agonists or inhibitors specific to proton-sensing channels, and a rapid development of potential anti-cancer drugs (Moraes, 1973; Yelshanskaya and Sobolevsky, 2022). Therefore, this review also summarizes and discusses the prospects of current drugs targeting proton-sensing channels or GPCRs in clinical trials, as well as potential cancer therapeutics. The aims of this paper are to deepen people’s understanding of the importance of proton-sensing ion channels or GPCRs in the occurrence and development of tumors; to make full use of their general and specific advantages as anticancer drug targets, and to develop more drugs targeting such channels or GPCRs for cancer treatment.

**TABLE 1:** Acid-sensitive ion channels/receptors.

Effect of H^+^	Way of activity	Ion channels/Receptors	H^+^ sensitivity range	Critical amino acids	Refs
Directly sense H^+^ and activate	Combined with H^+^ and activated	ASICs	4–7	Gly430	Zhang et al. (2022)
		TRPV1	<6	Glu600, Glu648	Dhaka et al. (2006), Zhang et al. (2021)
		P2X2	<7.5	His319	King et al. (1997), Clyne et al. (2002)
	The conformation changes after sensing H^+^ and binds to the G protein	OGR1	5.6–7.8	His245, His269	Rowe et al. (2021)
				Asp67, Glu149, Asp282	
		GPR4	5.6–7.6	His79, His165, His269	Rowe et al. (2021)
				Asp63, Glu145, Asp282	
		TDAG8	5.7–7.2	His243	Rowe et al. (2021)
				Asp60, Glu142, Asp286	
		G2A	6.6–8.2	His174	Rowe et al. (2021)
Indirectly sense H^+^ and activate	Activation of ASICs and TRP leads to membrane depolarization which triggers activation of VGCC	VGCC			Benarroch (2014), Milici and Talavera (2021), Zhou et al. (2023a), Sun et al. (2023)
	Proton-sensing GPCRs activate SOCC through the PLC/IP3/IP3R signaling pathway	SOCC			Jernigan et al. (2009), Wiley et al. (2019), Haque et al. (2020)

**TABLE 2:** Ca^2+^ signaling regulation by acidic pHe.

Ion channels/Receptors	Cell type	Effect of acidic pH on Channel’s/Receptors’ activity/Expression	Effect on Ca^2+^ signals		Effects on the tumor cells	Refs
ASICs	Breast cancer	Acidic microenvironment activates ASIC1/Ca^2+^ influx/[Ca^2+^]_i_ ↑/AKT/NF-κB/ROS			Inflammation	Gupta et al. (2016)
	Pancreatic Cancer	Acidic microenvironment activates ASIC1 and ASIC3/Ca^2+^ influx/[Ca^2+^]_i_ ↑/RhoA			Migration	Zhu et al. (2017)
TRPV1	HEK-293	E600 and E648 are key sites in the enhancement and activation of acid-induced channels mediated by TRPV1	Not assessed			Zhang et al. (2021)
	Dorsal Root Ganglion (DRG) Neurons	Mild acidosis activate TRPV1/Ca^2+^ influx/[Ca^2+^]i ↑				Heber et al. (2020)
	Synoviocytes	Acidic solution activate TRPV1/Ca^2+^ influx/[Ca^2+^]i ↑/ROS			Inflammation	Hu et al. (2008)
	PC-3	Not assessed	Ca^2+^/ERK1/2		Proliferation	Morelli et al. (2014)
	LNCaP	Not assessed	Ca^2+^/PI3K		Proliferation, Migration	Malagarie-Cazenave et al. (2009)
			Ca^2+^/ERK1/2			
	Colon cancer	Not assessed	Ca^2+^/NF-kB		Inflammation	Vinuesa et al. (2012)
			Ca^2+^/STAT3			
TRPV4	HEK-293	TRPV4 is gated by a drop of pH below 6 and the channel current reaches a maximum at a pH of about 4	Not assessed			Holzer (2011)
	Endometrial carcinoma	Not assessed	Ca^2+^/RhoA/ROCK1		Migration	Li et al. (2020)
TRPC1-TRPC4-TRPC5	HEK-293	The effects of changes in pH on TRPC4 and TRPC5 activity are the currents being increased by a reduction of pH down to about 6.5	Not assessed			Semtner et al. (2007)
	MDCK-F cell	Not assessed	Ca^2+^/MAPK Ca^2+^/PI3K/AKT		Migration, Proliferation	Fabian et al. (2008)
	MCF-7/ADM	Not assessed	Ca^2+^/CaMKKβ/AMPKA/mTOR		Proliferation	Zhang et al. (2017)
	CRC	Not assessed	Ca^2+^/β-catenin/ABCB1		Drug Resistance	Wang et al. (2015c)
TRPP2	HEK-293	TRPP2 is sensitive to acidic microenvironments	Not assessed			Holzer (2011)
	Hep2cell	Not assessed	Ca^2+^/CaMKKβ/AMPK/PERK/eIF2α		Migration	Liang et al. (2008), Wu et al. (2016)
VGCC	Melanoma	extracellular acidic pH/Ca^2+^ influx/[Ca^2+^]i ↑/NF-kB			Migration, Inflammation	Kato et al. (2007)
P2X2	HEK-293	Acidic microenvironment activates P2X2/Ca^2+^ influx/[Ca^2+^]i ↑				King et al. (1997), He et al. (2003), Stojilkovic et al. (2014)
	Walker 256	Not assessed	Ca^2+^/NF-kB		Migration, Inflammation	Zhang et al. (2020)
SOCC	HEK-293	Proton-sensing GPCRs activate SOCC through the PLC/IP3/IP3R signaling pathway	Not assessed			Wiley et al. (2019), Haque et al. (2020)
	Pancreatic stellate cells	Not assessed	Ca^2+^/AKT		Proliferation	Radoslavova et al. (2021)
	Breast cancer	Not assessed	Ca^2+^/PI3K/AKT/SgK1		Proliferation	Hasna et al. (2018)
	LCSC	Not assessed	Ca^2+^/CaN/NFAT		Drug Resistance	Wang et al. (2021)
OGR1	Medulloblastoma	human cerebellar granule cell tumor (medulloblastoma) cell line (DAOY) showed high levels of expression of the OGR1 and G2A, Extracellular Acidification/PLC/IP3/IP3R/[Ca^2+^]i ↑/MEK/ERK			Proliferation	Huang et al. (2008)
GPR4	Neuroblastoma (SH-SY5Y)	GPR4 mRNA expression was increased at both pH 6.4 and 7.4		Not assessed		Haque et al. (2020)
		Not assessed		H_2_O_2_/GPR4/Gq/PLC/IP3/IP3R/[Ca^2+^]i ↑/ROS	Inflammation	Haque et al. (2020)
TDAG8	HEK-293	TDAG8 regulates TRPV1 in acidic environments/[Ca^2+^]i ↑				Lin et al. (2017)
G2A	HEK-293	Co-expression of OGR1 and G2A promotes proton sensitivity and calcium influx and ER calcium release, G2A alone could not induce an increase in intracellular calcium concentration				Huang et al. (2016)

Although the influence of the acidic tumor microenvironment and proton-sensing GPCRs on tumor properties has been discussed by Justus et al. (2013) the role of calcium channels in specific tumors has also been well summarized (Marchi et al., 2020), there is still no paper that reviews the effect of proton-sensing ionchannels or GPCRs on tumor progression through the activation of the intracellular Ca^2+^ signaling pathway in response to the acidic tumor microenvironment. Therefore, we systematically examines, analyzes and summarizes this issue in the present paper.

## 2 Ion channels and tumors on the cell membrane that sense the acidic microenvironment and cause extrinsic calcium entry

### 2.1 Receptor-operated calcium channel (ROCC)

**FIGURE 1:**
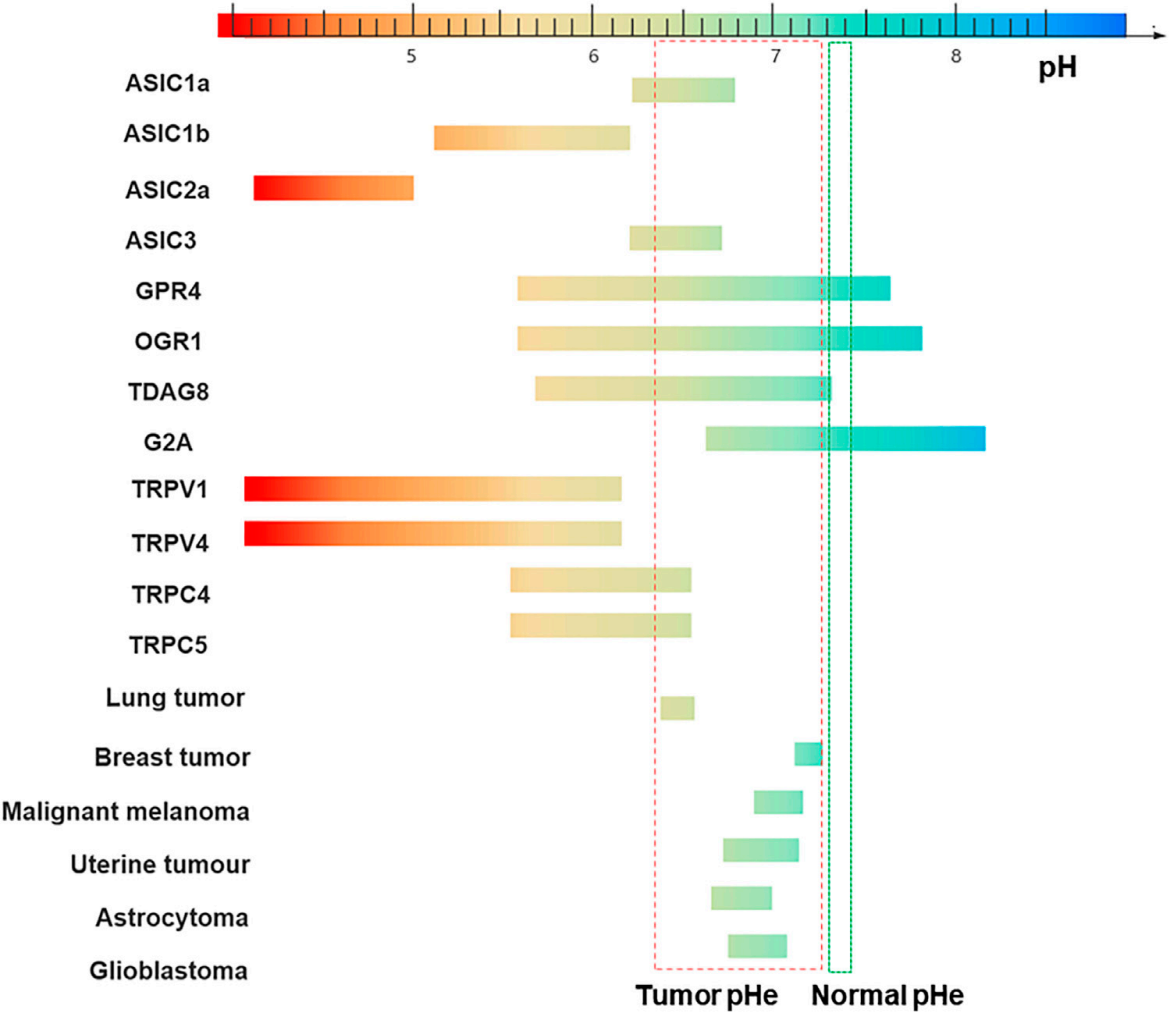
Sensitivity to pH of different channels/receptors and pH range of tumors. The pH-sensitive range of ASIC1a is 6.2–6.8, 5.1–6.2 for ASIC1b, 6.2–6.7 for ASIC3, and 4.1–5.0 for ASIC2a (Zhang et al., 2022). GPR4 is activated over a broad pH range from 5.6–7.6. The pH ranges of TDAG8, OGR1, and G2A are: 5.7–7.2, 5.6–7.8 and 6.6–8.2 (Sisignano et al., 2021). TRPV1 is gated open only if the extracellular pH is reduced below 6, in which case a sustained channel current is generated (Dhaka et al., 2006), TRPV4 is gated by a drop of pH below 6 and the channel current reaches a maximum at a pH of about 4. The effects of changes in pH on TRPC4 and TRPC5 activity are the currents being increased by a reduction of pH down to about 6.5 (Semtner et al., 2007; Holzer, 2011). The pHe of the different tumor cells: Lung tumor (6.4–6.5) Breast tumor (7.2–7.3) Malignant melanoma (6.9–7.1) Uterine tumour (6.7–7.1) Astrocytoma (6.6–7.0) Glioblastoma (6.7–7.0). Normal tissues extracellular pH (pHe): 7.3 to 7.4 (indicated in green) (Palmer et al., 1998). Tumor microenvironment (TME) pHe: 6.3 to 7.3 (indicated in red) (Pampus, 1963).

1) Acid-sensing ion channels (ASICs) are H^+^-gated cation channels, that are activated and open when the extracellular H^+^ concentration increases. When the extracellular pH drops rapidly from 7.4 to <6.9, the proton concentration gradient on both sides of the channel changes, and ASICs are activated (Figure 1). The activated ASIC exhibits cationic permeability, allowing the passage of Na^+^, Ca^2+^ and K^+^ (Na^+^ > Ca^2+^ > K^+^) (Zhang et al., 2022). There are four genes that are known to encode ASICs: ASIC1-4. The general structure of ASICs includes homotrimeric or heterotrimeric subgated channels. The overall structure consists of an intracellular N-terminus and an intracellular C-terminus, two transmembrane domains (TMs) that are voltage-independent and contribute to the recognition of extracellular ligands to regulate proton-gated currents (Ruan et al., 2021). Among them, the site of H^+^ stimulation as a receptor and related to channel gating is the extracellular glycine 430 near TM2. Regarding the pH sensitivity of ASIC subunits, different subunits have different pH sensitivity ranges. Mild extracellular acidosis can activate ASIC1 and ASIC3 channels, whereas ASIC2a requires strong acidic conditions for activation (Zhang et al., 2022). In addition, ASIC subunits have been detected in skin mechanosensitive receptors, dorsal root ganglion (DRG) innervating the colon and nerve endings in the aortic arch, which points to another function of ASICs as mechanosensors. However, the mechanism of mechanical gating of ASICs remains unclear. It was found that shear stress at pH 7.4 did not cause changes in the membrane current of oocytes expressing human ASIC. However, significant activation of ASIC channels and Ca^2+^ influx were observed under acidic conditions. This suggests that ASICs are indeed able to respond to mechanical forces and that the increase in ASIC activity by shear stress seems to depend on the acidic pH environment (Barth and Fronius, 2019).

**FIGURE 2:**
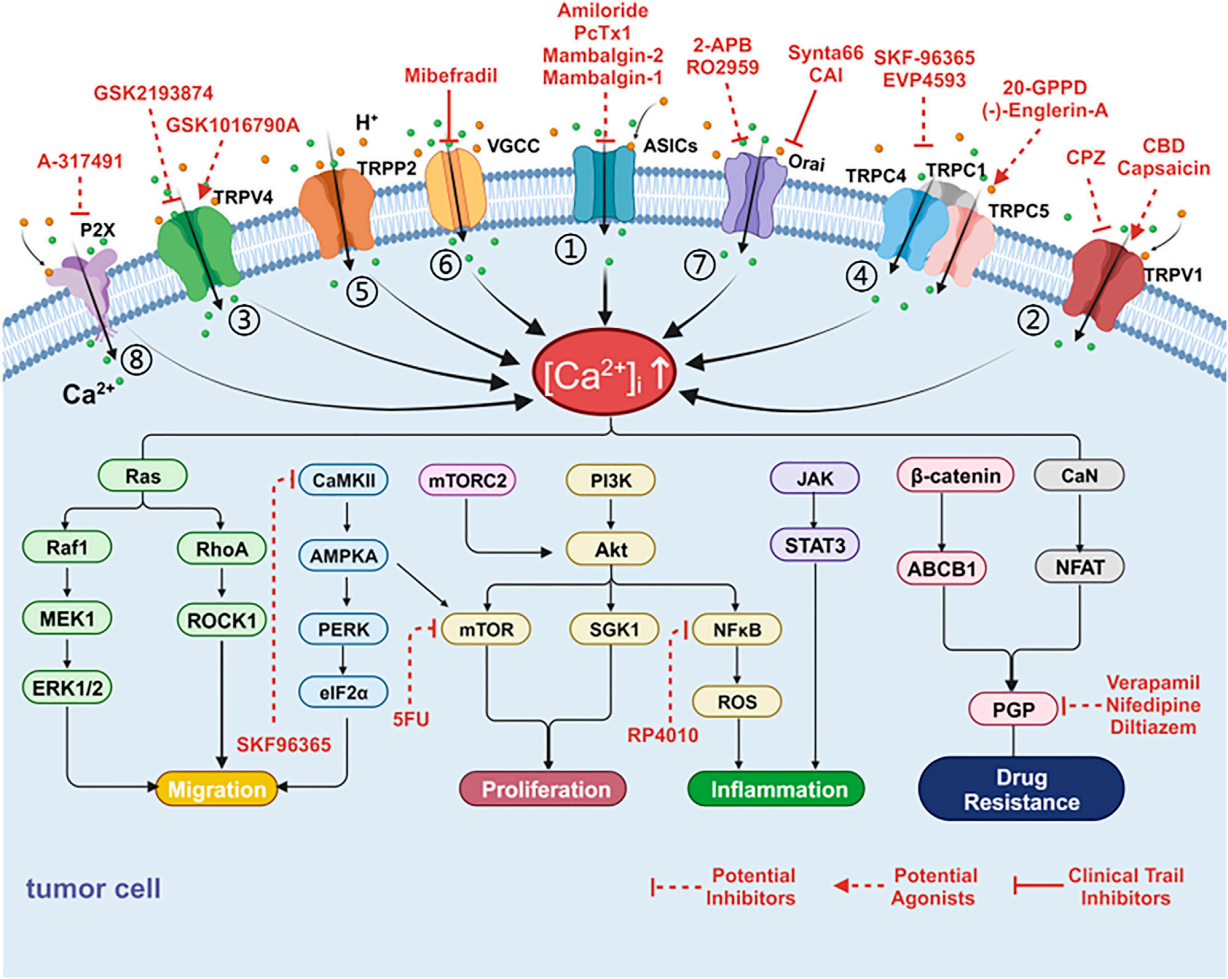
Cancer-associated alterations of Ca^2+^ fluxes at acidic tumor microenvironment and targets. Tumor cells can harness multiple alterations of Ca^2+^ fluxes at the plasma membrane in support of tumor progression or resistance to treatment. The channels for calcium inflow include acid-sensing ion channels (ASICs), transient receptor potential (TRP) channels, store operated calcium entry (SOCE), voltage-gated calcium channel (VGCC) and P2X receptors. These channels have been linked to proliferation, migration, inflammation, oxidative stress and superior resistance to drugs through various calcium related signaling pathways. (1) Ca^2+^ influx through ASIC activates AKT, NF-κB, and Ca^2+^/RhoA pathways to promote metastasis and produce inflammation and oxidative stress. (2) Activation of TRPV1 induces Ca^2+^ influx, which subsequently triggers ERK1/2, PI3K, NF-κB, and STAT3 signaling pathways to promote proliferation, migration, and inflammation. (3) TRPV4 and Ca^2+^ promote tumor metastasis through the RhoA/ROCK1 pathway. (4) TRPC1 mediates Ca^2+^ entry to activate MAPK and PI3K/AKT signaling pathways. TRPC5 overexpression activates CaMKII/AMPKA/mTOR as well as β-catenin nuclear translocation, ABCB1 expression and leads to chemotherapy resistance. (5) TRPP2/AMPK/PERK/eIF2α signaling pathways involved in cell proliferation. (6) VGCC may promote tumor cell proliferation through mTORC2/AKT pathway. (7) Ca2+ entry through SOCE activates PI3K/AKT/SgK1 to promote migration and leads to drug resistance through the CaN/NFAT pathway. (8) P2X stimulation enhances the PI3K/Akt pathway and NF-κB activity. Several targeting drugs involve the following channels: (1) Drugs targeting ASICs, TRP family members, SOCE, VGCC and P2X receptors; (2) Inhibitors targeting signaling pathways (i.e., PI3K/AKT/mTOR, NF-κB, P-GP, and CaMKII) [Ca^2+^]_i_: intracellular free calcium concentration.

There is increasing evidence that ASIC is related to the proliferation, migration and invasion of malignant tumors (Figure 2). The inhibition of ASIC1 can arrest the cell cycle of glioma D54-MG cells in G0/G1 phase and reduce the accumulation of cells in S and G2/M phases (Rooj et al., 2012). ASIC1a is overexpressed in hepatocellular carcinoma tissues and is related to the development of the disease. Silencing ASIC1a expression can inhibit the migration and invasion of cancer cells (Jin et al., 2015). Breast cancer cells express ASIC1. The acidic microenvironment contributes to breast tumor invasion and metastasis, which can activate AKT and NF-κB through calcium influx and produce reactive oxygen species (ROS) (Gupta et al., 2016). ASIC1 and ASIC3 are mainly expressed on the membrane of pancreatic cancer cells and upregulated in pancreatic cancer tissues, and ASIC1 and ASIC3 promote acid-induced EMT of pancreatic cancer by activating the Ca^2+^/RhoA pathway, leading to the metastasis of pancreatic cancer (Zhu et al., 2017). However, on the contrary, ASIC2 can inhibit the proliferation and migration of glioma cells. In one study, the surface expression of ASIC2 protein was detected in normal astrocytes, but was completely absent in high-grade malignant gliomas (Berdiev et al., 2003). In further experiments, it was found that the increase in ASIC2 protein on the cell surface could reduce the proliferation and migration of glioma cells. In addition, vascular smooth muscle cell (VSMC) migration is important for angiogenesis and vascular remodeling after injury. Another study showed that ASIC2 is also involved in inhibiting VSMC cell migration (Grifoni et al., 2008). Similar to the abovementioned high-grade glioma cells, increased ASIC2 cell surface expression can inhibit platelet-derived growth factor-induced cell migration (Liu C. et al., 2016). In addition, knockdown of ASIC2a in C6 glioma cells can increase acidosis-induced cytotoxicity through intracellular calcium overload, and subsequently affect cell invasion and migration. Therefore, in contrast to ASIC1a, ASIC2a, may play a protective role against extracellular acidosis-induced injury in C6 cells. Further studies are needed to more fully characterize the role of each member of the ASIC family in cancer (Xu et al., 2016).

2) Transient Receptor Potential (TRP) Channels: Mammalian TRP channel proteins form six transmembrane (6-TM) cation permeation channels, which can be divided into six subfamilies based on their amino acid sequence homology: TRPC, TRPV, TRPM, TRPA, TRPP and TRPML (Ramsey et al., 2006). TRP channels are a group of relatively nonspecific cation channels, that are mainly located in the plasma membrane of animal tissues. These channels respond to a variety of heterogeneous stimuli, including endogenous and exogenous chemical mediators, physical stimuli such as mechanical force (stretch sensitive) and temperature (heat sensitive), and free cytosolic Ca^2+^ ions. Many TRP ion channels mediate calcium influx into cells. There are two possible mechanisms by which TRP channels drive enhanced intracellular calcium concentrations: calcium efflux from the endoplasmic reticulum (ER), or the stimulation of calcium channels in the plasma membrane (Kadio et al., 2016). Calcium signaling is associated with multiple TRP channels (Yang and Kim, 2020). Among many TRP channel units, TRPV1, TRPV4, TRPC4, TRPC5 and TRPP2 are particularly sensitive to acidic microenvironments (Holzer, 2011). Two negatively charged residues (i.e., E600 and E648) have been revealed via cryo-electron microscopy to be key sites for TRPV1-mediated acid-evoked channel potentiation and activation (Zhang et al., 2021). TRP channels can affect the proliferation, migration, inflammation, oxidative stress and drug resistance of tumor cells (Chen et al., 2023) (Figure 2).

#### 2.1.1 The effect of TRP channels on tumor cell proliferation and progression

Norepinephrine has been shown to induce calcium influx by activating TRPV1, which subsequently triggers extracellular signal-regulated kinase 1/2 (ERK1/2), PLC, and PKC pathways to promote the proliferation of human prostate tumor PC-3 cells (Morelli et al., 2014). Malagarie-Cazenave et al. (2009) found that capsaicin, a TRPV1 agonist, inhibited ceramide production and increased androgen receptor (AR) expression by activating the PI3K and p44/42 MAPK (Erk1/2) pathways, thereby promoting the growth of human prostate tumor androgen-responsive LNCaP cells. Several recent studies have investigated the effect of targeting TRP channels on the growth of pancreatic ductal adenocarcinoma (PDAC). TRPV1 has been found to be associated with the promotion of PDAC cell proliferation and migration. Inhibition of TRPV4 reduced PDAC cell proliferation and migration and inhibited tumor growth (Yang and Kim, 2020; Zhong et al., 2022; Wei et al., 2023). In addition, there is evidence that TRPC1 may be associated with the characteristics of a variety of cancers (Elzamzamy et al., 2020). Dyrda et al. reported that TRPC1 activation depends on STIM1-activated Icrac current activation (Dyrda et al., 2020). However, activation of STIM1 does not necessarily activate TRPC1. The transmembrane protein STIM1 interacts with ORAI1, which activates CRAC channels, which in turn activates Ca^2+^-selective Icrac currents. STIM1 interacts with TRPC1 to form the STIM-ORAI1-TRPC1 complex and activates the SOCC channel, which facilitates the formation of heterotetrames of the nonselective cation channels TRPC1, TRPC4, and TRPC5 to facilitate further calcium influx (Moccia et al., 2023). It has been shown that TRPC1 is not sufficient to form channels when expressed on it (Beech, 2013). TRPC1 does not form a Ca^2+^-permeable channel *per se* but is essential for isomer formation with other members of the TRPC subfamily (Storch et al., 2012). Calcium enters through SOCE and binds calmodulin, leading to the activation of the phosphatase protein calcineurin, which activates the transcription factor NFAT. Active NFAT translocates to the nucleus and regulates the expression of genes that promote proliferation, migration, and survival (Fabian et al., 2008). TRPP2 is a membrane-associated protein that regulates cell signaling and the intracellular calcium concentration. TRPP2 attenuates HEK293T cell proliferation by activating the AMPK/PERK/eIF2α signaling pathway (Liang et al., 2008)

#### 2.1.2 The effect of TRP channels on tumor cell migration and invasion

Recent data from mouse models of colitis suggest that TRPV4, which is expressed in both vascular endothelial cells and bone marrow-derived macrophages, plays an important role in colitis-associated tumorigenesis (Matsumoto et al., 2020). Proteomics and bioinformatics analysis showed that TRPV4 was involved in calcium influx and promoted endometrial cancer (EC) cell migration through the RhoA/ROCK1 pathway (Li et al., 2020). The VPAC/TRPV4/Ca^2+^ signaling axis has been confirmed to be related to gastric cancer and to promote cancer cell metastasis. VPAC1 (vasoactive intestinal peptide (VIP) receptor) triggers TRPV4 channels via the PLC/DAG/PKC signaling pathway (Tang et al., 2019). TRPC1-mediated calcium entry can also promote cell migration by activating the MAPK and PI3K/AKT signaling pathways (Fabian et al., 2008). Calmodulin-dependent protein kinase β (CaMKKβ) is the upstream kinase of AMPK. CaMKKβ activates AMPK by increasing intracellular Ca^2+^. Increased TRPP2 promoted the invasion and metastasis of Hep2 cells, and TRPP2 siRNA significantly inhibited ATP-induced Ca^2+^ release in Hep2 cells and cell invasion (Wu et al., 2016).

#### 2.1.3 TRP channels activate inflammation and oxidative stress

ROS not only plays an important role in cell apoptosis and necrosis, but also participates in intercellular signal transduction and affects gene expression, which leads to tumorigenesis. Under physiological conditions, ROS keep the redox system stable and play an important role in human physiological processes. Once this dynamic balance is disrupted, it will lead to a large amount of ROS generation, causing oxidative stress, causing damage to cells and, in severe cases, cell death. Increasing evidence has found that the vast majority of tumor cells have defects in the anti-oxidative stress system, and ROS plays an important role in both promoting apoptosis and inhibiting survival. Oxidative stress can cause DNA base changes, strand breaks, increased expression of proto-oncogenes and inactivation of tumor suppressor genes in cells, which is closely related to the occurrence and development of many tumors (Yang et al., 2023).

Many recent studies in human tumors and cell lines have shown high expression of redox TRP channels, which are sensors of oxidative responses in cancer cells. In recent years, specific TRPS activated directly or indirectly by ROS have been named redox-sensitive TRP channels. The interaction between oxidative stress, TRP channels, and inflammation and their specific mechanisms for the tumorigenic process have been described in detail (Piciu et al., 2023). Redox TRP channels can be activated by ROS and affect cancer progression through different signaling pathways. For example, the activation of TRPV1 and TRPV4 in lung cancer cells induces Ca^2+^ influx, and eventually leads to tumor cell proliferation, migration and apoptosis by stimulating the MAPK pathway to promote cell apoptosis and increase ROS production. On the other hand, ROS activates NF-κB and STAT signaling pathways, Vinuesa et al. showed that TRPV1^−/−^ mice are more susceptible to dextran sodium sulfate-induced colon cancer. Their study showed that the NF-kB and STAT3 signaling pathways were hyperactivated in TRPV1^−/−^ mice, leading to the upregulation of a set of inflammatory factors (including IL-1 and IL-6) and invasive factors (such as MMP9), which subsequently enhanced carcinogenesis (Vinuesa et al., 2012).

#### 2.1.4 TRP channels induce drug resistance

Despite advances in specific therapies targeting various tumors in recent decades, treatment failure and mortality rates remain high. The development of drug resistance is a major challenge in cancer treatment. It was suggested that overexpression of TRPV1 could induce 5-FU resistance, improve the efficiency of DNA repair, and inhibit cell apoptosis. In this context, TRPV1 overexpression appears to activate the p38 MAPK signaling pathway and promote cell survival (Marchi et al., 2020). In adriamycin-resistant breast cancer cells MCF-7/ADM, adriamycin treatment increased TRPC5 expression, leading to the activation of the calmodulin-dependent signaling pathway (CaMKII/AMPKA/mTOR) and chemotherapy resistance (Zhang et al., 2017). In addition, multidrug resistance (MDR) is the main cause of chemotherapy failure, especially during the treatment of malignant tumors. MDR is the low sensitivity of specific cells to cancer chemotherapy-related drugs. A typical mechanism leading to MDR and chemotherapy failure is the overexpression of the permeability glycoprotein (P-gp) encoded by the ATP-binding cassette subfamily B member 1 (ABCB1). Ca^2+^ influx and aberrant Wnt/β-catenin signaling activation increase ABCB1 production, Overexpression of TRPC5 increases intracellular Ca^2+^, nuclear translocation of β-catenin, and expression of ABCB1 and leads to chemoresistance to 5-FU (Wang T. et al., 2015). TRPC5 overexpression also increases the expression of P-gP in the NFATC3-dependent pathway, leading to changes in doxorubicin localization and chemotherapy resistance (He et al., 2014). Moreover, TRPC5 channel expression was increased together with P-gp in breast cancer cell lines, and inhibition of TRPC5 reduced P-gp level and reversed cell drug resistance (Ma et al., 2012).

### 2.2 Voltage-gated calcium Channel (VGCC)

VGCCs are a group of voltage-gated ion channels found in the membrane of excitable cells such as muscle, glial cells, and neurons. VGCC activity is affected by changes in membrane voltage and is permeable to Ca^2+^. At physiological or resting membrane potentials, VGCCs are usually closed. They are activated at a depolarized membrane potential. VGCC can be divided into T, L, N, R, and P/Q subtypes, and their expression varies according to cell type (Catterall, 2011). Previous studies have demonstrated that the transient increase in intracellular free calcium concentration in response to extracellular acidic pH can be mediated by VGCC (Kato et al., 2007). Activation of ASICs and TRP channels leads to membrane depolarization that triggers VGCC activation (Benarroch, 2014; Milici and Talavera, 2021; Zhou R. P. et al., 2023; Sun et al., 2023).

However, VGCC changes have also been detected in malignant nonexcitatory cells (Figure 2). The results of a meta-analysis in 2015 showed that VGCC mRNA expression was upregulated in cancer tissues compared to normal tissues. L-type and T-type Ca^2+^ channels are two major VGCC channels that are aberrantly expressed in different tumors and are associated with tumor cell proliferation, migration, and anti-apoptosis (Wang C. Y. et al., 2015). For example, L-type calcium channels are significantly upregulated in colon cancer and esophageal cancer. The upregulation of T-type calcium channels has mainly been observed in prostate cancer, breast cancer and ovarian cancer. Such alterations have also been found in melanoma, retinoblastoma, glioma, glioblastoma, hepatocellular carcinoma, colon cancer, and esophageal cancer cells. On the other hand, members of the VGCC family are expressed at detectable levels in melanoma cells, but not in untransformed melanocytes, and cell cycle arrest is induced by the use of T-type channel inhibitors, with a significant increase in the percentage of cells in G1 phase and a decrease in S phase. Treatment with the T-type Ca^2+^ channel blocker Mibefradil was able to reduce esophageal and colon cancer cell proliferation and tumor growth in xenograft models of glioblastoma and ovarian cancer (Cui et al., 2017). In addition, it promotes the apoptosis of tumor cells by inhibiting the mTORC2/AKT pathway (Valerie et al., 2013). Recently, the L-type VGCC blocker verapamil revealed that inhibition of Ca influx mediated by L-type voltage-gated calcium channels was able to inhibit the collective migration and invasion of ameloblastoma (Li et al., 2022).

### 2.3 P2X receptors

P2X receptors (P2XRs) are ATP-gated ion channels present on the plasma membrane of most excitable and nonexcitable cells (North, 2002). ATP at appropriate concentrations has been shown to activate P2X7R and mediate the inward flow of Ca^2+^. Naemsch et al. (2001) To date, seven isoforms (i.e., P2XR1-7) have been cloned from mammalian cells, showing a broad affinity for ATP. Among the various P2X subunits, P2X1, P2X2, P2X3, P2X4, P2X5 and P2X7 are capable of being regulated by changes in pHe (Holzer, 2003). In general, P2XRs are inhibited by acidification; however, in contrast, extracellular acidification activates P2X2, reaching its maximum response to ATP at pH 6.5 (King et al., 1997; Stojilkovic et al., 2014). Moreover, acidification increases the amplitude of Ca^2+^ influx in P2X2R-expressing cells (He et al., 2003). Mutational analysis suggests that His319 is particularly important for the ability of protons to enhance the P2X2 receptor (Clyne et al., 2002).

During cancer development, ATP concentrations in the tumor microenvironment are high enough to activate P2X purinergic receptors. As early as 1976, Landry and Leininger showed that eATP, increased the permeability of Ehrtzman ascites tumor cells to extracellular Ca^2+^ (Landry and Lehninger, 1976). P2X2 receptor subunits are coexpressed with other P2X subtypes in many cell types, and several studies have implicated P2X2/3R in bone cancer pain perception (Mai et al., 2021). Moreover, in head and neck squamous cell carcinoma, cancer cells release large amounts of ATP, which activates P2X2 and P2X3 receptors in trigeminal ganglion neurons and enhances pain (Ye et al., 2014). The activation of P2X2/3R on the cell membrane causes calcium influx, which can further activate different intracellular signaling pathways (such as NF-kB and PKA/PKC), induce the production and release of damage factors, and trigger inflammatory responses that cause pain (Zhang et al., 2020). Substantial *in vitro* and *in vivo* evidence suggests that P2X7R mediates tumor nutrition/growth promotion. P2X7R antagonists can inhibit tumor growth and metastasis formation, and P2X7R expression or activation increases NFAT and NF-κB activity, intracellular Ca^2+^ levels and ATP production in addition to activating the c-Myc oncogene. In neuroblastoma cells, P2X7R stimulation enhances the PI3K/Akt pathway and decreases GSK3β activity. These effects were reversed by P2X7R antagonists (Di Virgilio et al., 2021) (Figure 2). A recent study showed that cancer cells express higher eATP synthase levels to produce extracellular ATP, thus stimulating the secretion of extracellular vesicles (EVs) by enhancing P2X receptor-triggered Ca^2+^ influx. EVs are important regulators in the tumor microenvironment that regulate immune cell populations and reduce antitumor response signaling (Kao et al., 2023).

### 2.4 Store-operated Ca^2+^ Channels (SOCC)

**FIGURE 3:**
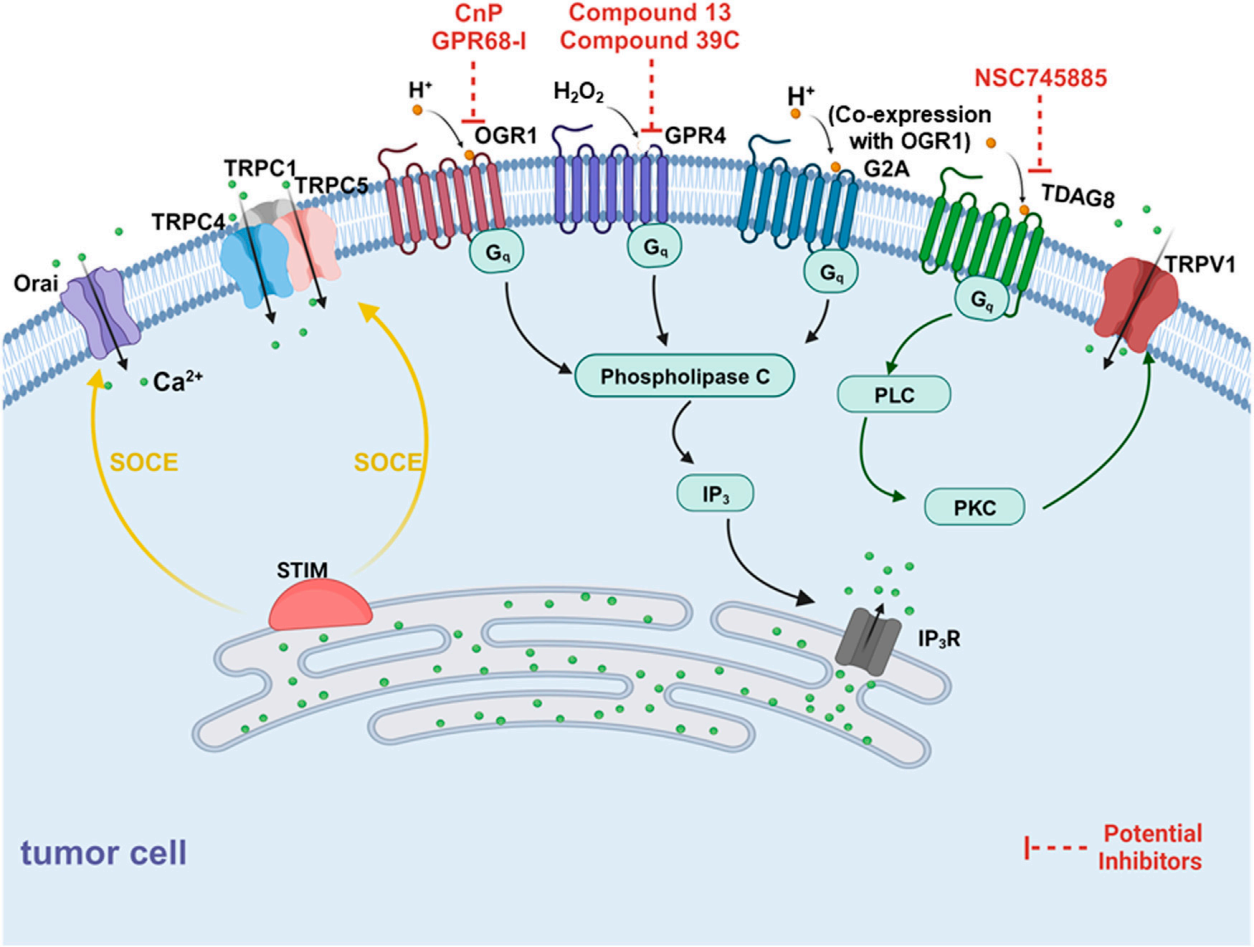
Schematic diagram of mechanisms for the extracellular acidification inducing alterations of Ca^2+^ fluxes. There are two components for Ca^2+^ elevation in response to elevated external protons, one is proton-sensing G-protein coupled receptors (GPCRs) and another is the store-operated Ca^2+^ entry pathway (SOCE). On one side, OGR1, G2A (co-expression with OGR1) and GPR4 (activated by H_2_O_2_) mediates mobilization of intracellular Ca^2+^ from SR via PLC-IP3-IP3R signaling pathway. Besides, TRPV1 can be activated by TDAG8 and mediate extracellular Ca^2+^ entry. On the other side, SOCE is regulated by H^+^ binding to proton-sensing GPCRs, activating phospholipase C (PLC) via Gq signaling, resulting in the production of IP3. IP3 depletes Ca^2+^ stores from the ER through the IP3R which is sensed by STIM1. STIM molecules multimerize forming puncta and translocate to co-assembling with the CRAC channel subunits ORAI1, activating the Ca^2+^ selective Icrac currents. Further, STIM1 forms the STIM1-ORAI1-TRPC1 directly interacting with TRPC1, TRPC4, and TRPC5 to induce Ca^2+^ entry.

SOCC is a kind of cell membrane calcium channel, and its opening is closely related to the release of intracellular calcium stores, which play an important role in both excitatory and nonexcitatory cells. The emptying of endoplasmic reticulum (ER) calcium stores activates Ca^2+^ channels and allows Ca^2+^ to enter the cytoplasm through the cell membrane in a process known as store-operated Ca^2+^ entry (SOCE). The activation mechanism of SOCC is mainly through the activation of phospholipase C (PLC) after the activation of receptors on the cell membrane. IP3 binds to the IP3 receptor on the intracellular Ca^2+^ pool, causing the release of calcium ions from the intracellular Ca^2+^ pool into the cytoplasm, leading to the depletion of the intracellular Ca^2+^ pool and triggering a large influx of calcium ions. The major SOCE participants are STIM1 and ORAI1. After Ca^2+^ depletion from ER stores, STIM1 interacts with ORAI1 and leads to the opening of Ca^2+^ release-activated channels (CRAC). Thus the influx of Ca^2+^ is allowed (Zhang et al., 2005; Feske et al., 2006; Stathopulos et al., 2006; Stathopulos et al., 2008; Stathopulos and Ikura, 2017) (Figure 3). As one of the main pathways for calcium entry, SOCE plays an important role in intracellular calcium homeostasis. Although there is no direct evidence that SOCC can directly sense the acidic extracellular microenvironment, previous studies have shown that ASIC1 can activate SOCE in pulmonary arterioles (Jernigan et al., 2009). And ASIC1 enhances SOCE after exposure to chronic hypoxia (CH) (Jernigan et al., 2012). It was subsequently determined that ASIC1 contributes to the development of chronic hypoxia (CH) and acute hypoxic pulmonary vasoconstriction (HPV) -induced pulmonary hypertension (PH) through the activation of SOCE (Nitta et al., 2014). A recent study has shown that acidosis activates SOCE by activating ASICs in vascular smooth muscle cells (VSMCs). It was also observed that the SOCE of VSMCs at lower pH values was ASIC3 dependent (Rehni et al., 2019). In addition, proton-sensing GPCRs (detailed below) can also activate SOCC through PLC-IP3-IP3R signaling.

Many studies have found that SOCE is closely related to the progression of various cancers (Pan and Ma, 2015) (Figure 2). Overexpression of Orai1 and STIM1 is associated with increased SOCE in ovarian cancer cells and appears to be associated with an Akt-dependent pathway (Schmidt et al., 2014). Recent studies have shown that Orai1 channels regulate pancreatic stellate cell proliferation and transforming growth factor (TGF) secretion through the AKT signaling pathway, and TGF induces a higher SOCE response and promotes AKT phosphorylation and cell proliferation by increasing Orai1 (Radoslavova et al., 2021). Orai3 overexpression in breast cancer is associated with chemotherapy resistance in patients and cell lines. Orai3 acts through the PI3K/SGK1 pathway to inhibit paclitaxel-, cisplatin-, and 5-FU-induced apoptosis (Hasna et al., 2018). SGK1 stimulates cell proliferation and confers cell survival, thus actively participating in the regulation of tumor growth (Schmidt et al., 2014). High expression of STIM1 and increased SOCE lead to increased activity of nuclear factor of activated T cells (NFAT) (Kutschat et al., 2021). Interestingly, it has recently been shown that STIM1 regulates breast cancer cell migration through NFAT1 signaling independent of Orai1 and SOCE in breast cancer cells (Hammad et al., 2023). High levels of FGF19 in HCC patients can promote the self-renewal of liver cancer stem cells (LCSC) by activating the SOCE/NFATc2 pathway (Wang et al., 2021). Inhibition of calcium signaling prevents exhaustion and enhances the antileukemic efficacy of CAR-T cells, thus making it an effective therapy for tumor recognition and elimination by inhibiting the SOCE-calcineurase-NFAT pathway (Shao et al., 2022). SOCE can also affect the proliferation and migration of breast cancer cells induced by inflammatory signaling (Alqinyah et al., 2023).

## 3 Proton-sensing G-protein coupled receptors (GPCRs) at the cell membrane and tumors

With seven transmembrane domains, GPCRs are the largest family of membrane protein receptors in the human genome and the genomes of many other species. The GPCRs family is also the target family of the most approved drugs (Hauser et al., 2017; Sriram and Insel, 2018; Eiger et al., 2022). Proton-sensing G-protein coupled receptors (GPCRs) belong to the GPCRs subfamily and were first identified in 2003 by Ludwig et al. The family consists of four members: GPR4, TDAG8 (GPR65), OGR1 (GPR68), and G2A (GPR132) (Ludwig et al., 2003; Hu et al., 2017; Sisignano et al., 2021). They can sense the extracellular acidic microenvironment and activate G proteins, thereby regulating multiple intracellular signaling pathways (Figure 1). This family has also been implicated in various biological processes and diseases, including ischemia, inflammation and cancer (Rowe et al., 2021). Notably, there is growing evidence showing that proton-sensing GPCRs affect tumor progression by sensing the acidic microenvironment of tumor cells and regulating multiple intracellular signaling pathways, including the calcium signaling pathway (Table 3).

**TABLE 3:** Effects of proton-sensing GPCRs on traits of the cancer cells.

Proton-sensing GPCRs	Proliferation	Migration	Angiogenesis	Immune escape
OGR1	Activator	Activator/Inhibitor	Activator	Activator
GPR4	Activator	Activator/Inhibitor	Activator	—
TDAG8	Activator/Inhibitor	Inhibitor	—	Activator
G2A	Inhibitor	Activator	—	—
GPR31	Activator	Activator	—	—
GPR151	—	—	—	—

—: No reports related.

### 3.1 OGR1

OGR1 was originally cloned and characterized from human ovarian cancer tissue (it is not expressed in healthy ovarian tissue) (Xu and Casey, 1996), and its role as a proton-sensing GPCR was first described by Ludwig et al. (2003) The author observed the protons in the largest activation OGR1 at a pH of 6.8. OGR1 is considered to be activated by cell stretching and acidosis mechanoreceptor (Wei et al., 2018). OGR1 expression is most abundant in the pituitary gland, followed by the esophageal mucosa, cerebellum, and lung. In addition, OGR1 is usually expressed to varying degrees in most human tissues. OGR1 has been reported to sense the extracellular acidic environment and induce a significant increase in intracellular Ca^2+^ concentration (Ludwig et al., 2003; Seuwen et al., 2006; Huang et al., 2008). Many studies have shown that OGR1 protein binds to extracellular protons through its exposed histidine residues and is then activated. Through the G_q/11_/PLC/PI3 signaling pathway, OGR1 activates the PI3R channel on the endoplasmic reticulum and releases Ca^2+^ from the endoplasmic reticulum, leading to an increase in the intracellular Ca^2+^ concentration (Ludwig et al., 2003; Ichimonji et al., 2010; Liu et al., 2010). Meanwhile, the release of Ca^2+^ from the ER may also activate SOCC and cause changes in intracellular calcium signaling (Xu et al., 2018).

Previous studies have reported that an acidic microenvironment regulates the occurrence and development of tumors through the OGR1/Ca^2+^ signaling pathway (Figure 3). Two signaling pathways may be involved in this regulation: i) The OGR1/Gq/PLC/Ca^2+^/MEK/ERK pathway: Huang et al. demonstrated that OGR1 can activate gene transcriptional pathways in response to Ca^2+^ release from intracellular Ca^2+^ stores, thereby activating the MEK/ERK pathway and participating in tumor formation (Huang et al., 2008). ii) The OGR1/TRPC4/Ca^2+^ pathway: Wei et al. (2017) demonstrated that activation of OGR1 promoted TRPC4 expression and led to the opening of TRPC4 channels, which resulted in Ca^2+^ influx and enhanced the migration of medulloblastoma cells. However, whether other signaling pathways are involved in this regulatory process has not been reported. The molecular mechanisms by which the extracellular acidic environment of tumor cells activates the OGR1/Ca^2+^ signaling pathway and their relationship to tumor behavior require further investigation.

A large amount of evidence shows that the expression level of OGR1 is closely related to tumor proliferation, metastasis, angiogenesis and tumor immunity. i) OGR1 promotes tumor cell proliferation and tumor growth in prostate cancer (Singh et al., 2007), melanoma, colorectal cancer and pancreatic ductal adenocarcinoma (Li et al., 2009; Horman et al., 2017; Wiley et al., 2018; Cao et al., 2021). Moreover, OGR1 mRNA expression is upregulated in many tumors (Wiley et al., 2019). ii) In contrast to the promoting role of OGR1 in tumor proliferation, OGR1 has been demonstrated to be a tumor metastasis suppressor gene in prostate cancer (Singh et al., 2007) and ovarian cancer (Ren and Zhang, 2011) OGR1 expression was lower in distant metastatic lesions than in primary tumors (LaTulippe et al., 2002). iii) Li et al. (2009) reported that OGR1 knockout (KO) reduced angiogenesis in melanoma cells. Melanoma cell tumor vessels were significantly reduced in OGR1 KO mice compared to WT mice, indicating reduced angiogenesis in KO mouse tumors. iv) OGR1 is involved in tumor immunity. OGR1 expression in bone marrow-derived cells was found to be needed for immunosuppression induced by prostate cancer cells (Yan et al., 2014). A recent study also showed that the highly acidic microenvironment of melanoma induces OGR1 expression in T cells, impairs its effective function, contributes to immune escape and promotes tumor growth (Cao et al., 2021). However, the function of this receptor, especially in response to the acidic tumor microenvironment and the molecular mechanisms that alter the biological characteristics of multiple types of tumor cells, remains elusive. Therefore, more studies are needed to evaluate the role of OGR1 in tumorigenesis and progression.

### 3.2 GPR4

The GPR4 receptor is widely expressed in different tissues, it is activated in the pH range of 5.6–7.6; is involved in the inflammatory response and angiogenesis; and is overexpressed in tumors. GPR4 can be activated by hydrogen peroxide (H_2_O_2_) to promote the release of Ca^2+^ from endoplasmic reticulum (ER) storage in the cytoplasm through the G_q_-mediated intracellular signaling pathway (Haque et al., 2020). However, there is a lack of evidence that GPR4 is activated by extracellular acid to promote the release of ER calcium through the G_q_/PLC/IP3/IP3R pathway. Further studies are needed to confirm whether GPR4 can affect tumor progression by changing calcium signaling.

Many studies have shown that GPR4 is closely related to tumors. This is mainly reflected in the following two aspects. i) GPR4 is highly expressed in human tumors and promotes the proliferation of tumor cells (Sisignano et al., 2021) and angiogenesis in an acidic microenvironment (Wyder et al., 2011; Jing et al., 2016b). For example, in orthotopic tumor models of breast and colon cancer cells, GPR4 is activated after pH reduction and promotes tumor growth and pathological angiogenesis through p38-mediated secretion of interleukin-6 (IL-6), IL-8, and vascular endothelial growth factor-A (VEGF-A). Conversely, GPR4 knockout mice showed inhibited tumor growth (Wyder et al., 2011; Jing et al., 2016b). In addition, GPR4 is also known to play an important role in ovarian cancer growth and angiogenesis (Bai et al., 2021). ii) The role of GPR4 in cancer metastasis is controversial. For example, in ovarian cancer cells, downregulation of GPR4 inhibits cancer cell invasion, thus confirming that GPR4 plays a promoting role in ovarian cancer cell invasion. A recent study also showed that GPR4 is involved in the migration of melanoma cells and is enhanced in the range of pH 6.5 to 7.5, suggesting that an acidic microenvironment may promote melanoma invasion and metastasis through GPR4 (Bai et al., 2021; Stolwijk et al., 2023). However, the opposite result was found in another study, when GPR4 was overexpressed in mouse B16F10 melanoma cells, acidosis-activated GPR4 inhibited cancer cell migration and reduced melanoma cell lung metastasis (Castellone et al., 2011; Zhang et al., 2012). This may be because unlike Judith et al., who exposed GPR4-overexpressing melanoma cells to relatively normal pHe levels (6.5–7.5), Justus et al., who studied murine B16F10 melanoma cells only at very low pHe levels (6.4) and reported that cell migration is correlated with the strength of cell-matrix interactions. pHe affects human melanoma cell migration by modulating cell-matrix interactions. If the interactions are too strong [i.e., the pHe is too low (6.5)] or too weak [i.e., the pHe is too high (7.5)], melanoma cell migration is maintained (Stock et al., 2005). In summary, current evidence supports that GPR4 promotes tumor proliferation and angiogenesis. However, the effect of GPR4 on tumor invasion and metastasis may act as both a tumor metastasis promoter and a tumor metastasis suppressor, which may be related to tumor cell type. These observations also suggest that GPR4 senses acidity and promotes downstream signaling, which is highly dependent on the environment and cell specificity, and the role of the extracellular acidic environment of different tumor cells in GPR4 and their relationship to tumor behavior requires further investigation.

### 3.3 TDAG8

Unlike other proton-sensing GPCRs, TDAG8 is almost exclusively expressed in lymphoid tissues (Radu et al., 2006). A recent study showed that TDAG8 enhances acid-induced calcium influx by regulating TRPV1 (Figure 3) (Haque et al., 2020). TDAG8 may also affect tumor growth, migration, and apoptosis evasion. i) The role of TDAG8 in tumor growth remains controversial. Overexpression of TDAG8 in Lewis lung cancer (LLC) has been reported to enhance tumor growth via PKA and extracellular signal-regulated kinase (ERK) (Ihara et al., 2010). In contrast, TDAG8 expression in U937 lymphoma cells at acidic pH suppressed the expression of the oncogene c-Myc, thereby inhibiting tumor growth (Justus et al., 2017). As mentioned above, it is possible that TDAG8 has completely opposite effects on tumor growth characteristics due to different tumor cell types. ii) TDAG8 has a potential role in inhibiting tumor migration in lymphoma. TDAG8 gene expression was significantly reduced compared with that in normal immune cells and tissues rich in leukocytes. Functional studies showed that TDAG8 inhibited the migration and metastasis of U937 cancer cells under conditions of extracellular acidosis (Justus et al., 2017). iii) TDAG8 has also been shown to promote apoptosis evasion in lymphoma cells under glutamine starvation by activating the MEK/ERK pathway (Ryder et al., 2012). iv) Potential anti-inflammatory effects of TDAG8: A recent study showed that TDAG8 knockout exacerbated intestinal inflammation in mice. In addition, TDAG8 knockout aggravated colitis-associated colorectal tumorigenesis in mice. This suggests that enhanced TDAG8 expression may have anti-inflammatory therapeutic effects on inflammatory bowel disease (IBD) and reduce the risk of colitis-associated CRC (Marie et al., 2022).

### 3.4 G2A

G2A is mainly expressed in leukocytes and is associated with the migration of macrophages. In contrast to the other three GPCRs, G2A does not exhibit robust pH-dependent signaling and exhibits the weakest proton sensitivity of the four receptors, with a pH range of 6.6–8.2. A study showed that G2A did not increase intracellular calcium levels under moderate acid stimulation (pH 6.0), while coexpression of OGR1 and G2A enhanced proton sensitivity and intracellular calcium levels in HEK293T cells. It is possible that calcium influx is promoted by upregulating the activity of G_s_/PKA/cAMP channels (Huang et al., 2016).

G2A is widely expressed in a variety of tumor cells (Insel et al., 2020), and its effects on tumors mainly include three aspects: i) G2A has a potential inhibitory effect on hematological cancers. A recent study found that activation of G2A by 8-gingerol (8 GL) as an agonist can induce cell differentiation and inhibit tumor growth in acute myeloid leukemia (AML) (Yi et al., 2022). ii) G2A promotes tumor cell metastasis. In the tumor microenvironment, G2A on macrophages can sense and respond to lactate signals from cancer cells, thereby promoting cancer cell adhesion, migration and invasion (Chen P. et al., 2017). iii) The oncogenic transformation potential of G2A is controversial. It was initially found that overexpression of G2A in RAT-1 rat fibroblasts attenuated the transforming potential of BCR-ABL and other oncogenes (Weng et al., 1998). Later studies by Le et al. in mouse leukemia models also confirmed this view (Le et al., 2002). However, in NIH3T3 fibroblasts, G2A can enhance the oncogenic transformation of fibroblasts, leading to increased tumorigenicity in mice (Zohn et al., 2000). In conclusion, G2A has obvious oncogenicity in fibroblasts and can promote tumor cell proliferation. In contrast, coexpression of G2A and BCR-ABL inhibited the ability of the highly activated tyrosine kinase. This suggests that coexpression of G2A and BCR-ABL may somehow disrupt cell cycle regulation and thus inhibit proliferation. Moreover, in contrast to its oncogenic role in fibroblasts, G2A in lymphocytes inhibits the leukemogenic potency of BCR-ABL by activating RhoA to alter cytoskeletal organization and cell adhesion and regulate cell migration behavior.

### 3.5 Other potential proton-sensing GPCRs: GPR31 and GPR151

In addition to the four known proton-sensing GPCRs described above, the orphan receptors GPR31 and GPR151 were recently found to be activated under acidic conditions *in vitro* recently. They sense pH in a range of: pH 5-6, with maximal activity at pH 5.8. At least three residues on the GPR31 and GPR151 proteins are involved in the proton sensing process (Mashiko et al., 2019). When GPR31 and GPR151 are activated by protons, they can form fusion proteins with G_i_, thereby regulating their downstream signaling pathways. For example, GPR151 has been reported to work through the G_i_/ERK pathway, resulting in ERK-dependent neuroinflammation (Jiang et al., 2021).

GPR31 and GPR151 are closely related to tumorigenesis and development. Recently, GPR31 was reported to be associated with prostate cancer progression (Honn et al., 2016). GPR31 has also been reported to regulate the membrane association of KRAS and play an important role in KRAS-dependent tumor survival and proliferation (Fehrenbacher et al., 2017). *In vitro* studies also demonstrated that 12-HETE enhanced hepatocellular carcinoma (HCC) cell migration by inducing epithelial-mesenchymal transition (EMT) via activation of GPR31 (Yang et al., 2019). GPR151 is reported to be expressed specifically in the habenular area of the nervous system of vertebrates (Broms et al., 2015) and is also highly expressed in both peritoneal carcinomatosis of colorectal cancer (Breitenbuch et al., 2023) and squamous cell carcinomas (Förch et al., 2021). GPR31/151 may be involved in the regulation of calcium signaling. Evidence shows that 12 (S)-HETE inhibits adenylyl cyclase (AC) activity through GPR31 and leads to Ras-associated protein 1 (Rap1) and p38 activation, as well as low but detectable calcium flow (Van Doren et al., 2021). Overexpression of GPR151 significantly increased the P2X3-induced increase in intracellular calcium concentration (Xia et al., 2021). However, it has not been reported that acidic activation of GPR31/151 induces an increase in the intracellular calcium concentration. Future research should examine whether protons regulate calcium signaling through GPR31/151 and play a role in tumor progression remains to be investigated.

Above all, proton perceptual GPCRs play an important role in the development of tumors but are worthy of attention in the following aspects: i) specific proton-sensing GPCRs have different effects on different tumor properties, e.g., G2A functions as both a metastasis promoter and a proliferation suppressor; ii) specific proton-sensing GPCRs have different effects on tumor homology properties: TDAG8 promotes and inhibits tumor proliferation in LLC and lymphoma cells, respectively; and iii) specific proton-sensing GPCRs have different effects on the same characteristics of the same type of tumor. Similarly, in melanoma cells, the effect of GPR4 on tumor cell migration was opposite in the two studies. These observations suggest that the role of proton-sensing GPCRs in sensing acidity and promoting downstream signaling is closely related to cell specificity, the tumor microenvironment, gene coexpression and protein-protein interaction (PPI). Therefore, it is necessary to conduct more in-depth studies to better elucidate the detailed effects and complex mechanisms of GPCRs on tumorigenesis and development, and lay the foundation for the development of anticancer drugs targeting such GPCRs.

## 4 Therapeutic implications: targeting Ca^2+^ signaling driven by acidic microenvironments

As described in this review, calcium signaling enhanced by the acidic tumor microenvironment is widely involved in cancer progression. These observations collectively suggest that Ca^2+^ signaling is a reliable target for novel anticancer therapies, providing a wealth of potential targets for drug modulation and cancer chemotherapy; thus, inhibition of Ca^2+^ signaling may be a promising strategy for cancer therapy. New drugs targeting calcium signaling include transient voltage-gated calcium channel inhibitors and Orai inhibitors. However, most of these drugs are still in the early stage of research and lack clinical application of calcium signaling anticancer therapy (Sharma et al., 2021). In this section, we attempt to summarize the known channels/receptors that target the above cancer-related calcium and that have been investigated in preclinical studies or have been in clinical trials, as well as drugs with promising cancer therapeutic capabilities (Table 4).

**TABLE 4:** Inhibitor and agonist targeting acid-sensing calcium ion channels/receptors.

Ion channels/Receptors		Compound	Mechanism	Disease/Cell	Clinical trial	Refs
ASIC	ASIC1a	Amiloride	Inhibitor	Gastroesophageal Reflux Disease	NCT01095133	Bulsiewicz et al. (2013)
		Amiloride, PcTx1	Inhibitor	Glioblastoma		Escoubas et al. (2000), Hegde et al. (2004)
		Mambalgin-2	Inhibitor	Glioma, Lung Adenocarcinoma		Bychkov et al. (2020a), Sudarikova et al. (2022)
		A-317567	Inhibitor	Dorsal Root Ganglion (DRG) Neurons		Dubé et al. (2005)
		NSAIDs	Inhibitor	COS cell		Voilley et al. (2001)
		MitTx, Big dynorphin	Agonist	HEK-293		Sherwood et al. (2012), Borg et al. (2020)
		2-guanidine-4-methylquinazoline (GMQ)	Agonist	CHO cell		Alijevic et al. (2018)
		FMRFamide-like neuropeptides, Arachidonic acid, Nitric oxide	Agonist	DRG Neurons		Xie et al. (2003), Cadiou et al. (2007), Smith et al. (2007)
		Spermine	Agonist	Ischemic Neuronal Injury		Duan et al. (2011)
	ASIC1b	Amiloride	Inhibitor	Glioma		Berdiev et al. (2003)
		GMQ	Agonist	CHO cell		Alijevic et al. (2018)
		Nitric oxide	Agonist	DRG neurons		Cadiou et al. (2007)
				CHO cell		
	ASIC2	Amiloride	Inhibitor	Glioma		Grifoni et al. (2008)
		Mambalgin-1	Inhibitor	Leukemia, Glioma, Melanoma		Bychkov et al. (2020a), Bychkov et al. (2020b), Bychkov et al. (2021)
		Nitric oxide	Agonist	DRG neurons		Cadiou et al. (2007)
				CHO cell		
	ASIC3	Amiloride	Inhibitor	Pancreatic Cancer		Zhu et al. (2017)
		ApeTx2	Inhibitor	COS cell		Diochot et al. (2004)
		NSAIDs	Inhibitor	Fibromyalgia		Han et al. (2023)
		A-317567	Inhibitor	Osteoarthritis		Kuduk et al. (2010)
		GMQ	Agonist	HEK-293		Sherwood et al. (2012)
		Guanabenz (GBZ), Sephin1, 4-Chlorophenylguanidine	Agonist	CHO cell		Agharkar and Gonzales (2017), Callejo et al. (2020)
		FMRFamide-like neuropeptides, Arachidonic acid, Nitric oxide	Agonist	DRG neurons		Xie et al. (2003), Cadiou et al. (2007), Smith et al. (2007)
TRP	TRPV1	Capsazepine (CPZ)	Inhibitor	Oral squamous cell carcinoma (OSCC), Prostate cancer		Sánchez et al. (2006), Gonzales et al. (2014)
		SB-705498	Inhibitor	Migraine	NCT00269022	Chizh et al. (2007)
		CBD	Agonist	Colon cancer		Seltzer et al. (2020)
		Capsaicin	Agonist	Renal cancer, Thyroid cancer, Prostate cancer, Breast cancer	NCT03794388	Liu et al. (2016b), Xu et al. (2020), Dupoiron et al. (2022), Sánchez et al. (2022)
	TRPV4	RN-9893, BTP2	Inhibitor			Lawhorn et al. (2020)
		GSK2193874	Inhibitor	Prostate cancer, Lung cancer		Zheng et al. (2019), Washiya et al. (2020)
		GSK2798745	Inhibitor	Heart Failure	NCT02119260	Goyal et al. (2019)
		GSK1016790A	Agonist	Breast cancer		Peters et al. (2017)
	TRPC	SKF-96365	Inhibitor	Glioblastoma		Bomben and Sontheimer (2008), Chigurupati et al. (2010), Ding et al. (2010), Wu et al. (2021)
		20-GPPD	Agonist	Colon cancer		Hwang et al. (2013)
	TRPC1- Orai- STIM1	EVP4593	Inhibitor	Neuroblastoma		Vigont et al. (2014)
	TRPC4/5	ML204	Inhibitor	human lung microvascular endothelial cells (HLMVEC)		Zhou et al. (2023b)
		(−)-Englerin-A	Agonist	Renal cancer		Akbulut et al. (2015), Seenadera et al. (2022)
VGCC	T-type	Mibefradil	Inhibitor	Ovarian cancer, Glioblastoma	NCT01480050	Li et al. (2011), Dziegielewska et al. (2016)
					NCT02202993	
		NNC-55–0396	Inhibitor			Kale et al. (2015), Kim et al. (2015)
	L-type	Verapamil, Nifedipine, Diltiazem	Inhibitor	Ovarian cancer, Breast cancer, Glioblastoma, Colorectal cancer, Lung cancer, Hepatocelluar carcinoma		Wu et al. (2021)
SOCE	CRAC	RP4010	Inhibitor	Esophageal cancer, Lymphadenoma	Phase I/Ib trial	Cui et al. (2018)
					NCT03119467	
		Synta66	Inhibitor	Chronic lymphocytic leukemia	NCT03294980	Debant et al. (2019)
		CAI	Inhibitor	Ovarian cancer, Lung cancer	Phase II clinical trial	Hussain et al. (2003), Mignen et al. (2005), Chen et al. (2017a)
	STIM1	ML-9	Translocation inhibitor	Prostate cancer		Kondratskyi et al. (2014)
	Orai1-STIM1	SKF96365	Inhibitor	Esophageal cancer, Breast cancer, Colon cancer		Jing et al. (2016a)
	Orai1-STIM2	RO2959	Inhibitor			Chen et al. (2013)
		2-APB and its analogues, DPB162-AE, DPB163-AE	Inhibitor	Colon cancer, Glioma, Breast cancer		Prakriya and Lewis (2001), Goto et al. (2010), Motiani et al. (2013), Wang et al. (2015b)
P2X	P2X2/3	A-317491	Inhibitor	Femoral neoplasms		Gu et al. (2023)
Proton-Sensing GPCR	OGR1	GPR68-I	Antagonist	Colitis		Sanderlin et al. (2019), de Vallière et al. (2021)
		3,5-disubstituted isoxazoles	Agonist	Notch-activated epicardium-derived cells (NECs)		Russell et al. (2012)
		Ogerin, lorazepam	Positive Allosteric Modulators			Chen et al. (2011), Huang et al. (2015)
		MS48107	Positive Allosteric Modulators	HEK-293		Yu et al. (2019)
	GPR4	Compound 13	Antagonist	Colitis		Velcicky et al. (2017)
		Compound 39c	Antagonist	Arthritis		Miltz et al. (2017)
		Compound 3b	Antagonist	Myocardial Infarction		Fukuda et al. (2016)
	TDAG8	NSC745885	Antagonist	Arthritis		Kung et al. (2020)
		ZINC62678696	Negative Allosteric Modulators			Huang et al. (2015)
		Psychosine	Agonist	HEK-293		Im et al. (2001)
		BTB09089	Agonist	Splenocytes		Onozawa et al. (2012)
	G2A	Lysophosphatidylcholine (LPC)	Antagonist	NIH-3T3		Murakami et al. (2004)
		Telmisartan, GSK1820795A	Antagonist	HEK-293		Foster et al. (2019)
		8 GL	Agonist	Human primary AML cells		Yi et al. (2022)
		9S-HODE, 11-HETE	Agonist	CHO cell		Obinata et al. (2005), Yin et al. (2009)
		N-palmitoylglycine, N-linoleoylglycine, ONC212, 11,12-EET, 9,10-EpOME	Agonist	HEK-293		Lahvic et al. (2018), Foster et al. (2019), Nii et al. (2019)

### 4.1 Current clinical trials of drugs

Mibefradil is a T-type and L-type calcium channel blocker used in antihypertensive treatment. Mibefradil was shown to be effective in reducing tumor size and improving survival in animal models of glioma (Kale et al., 2015). Therefore, Mibefradil has entered clinical trials for the treatment of glioma (Krouse et al., 2015). The CRAC channel blocker RP4010 was tested in a phase I/Ib trial in lymphoma, but was discontinued in 2019 due to safety concerns (Cui et al., 2018). In addition, a study conducted in chronic lymphocytic leukemia (CLL) also showed an anticancer effect of the CRAC channel blocker Synta66 (NCT03294980). (Debant et al., 2019).

### 4.2 Potential drugs for the treatment of tumors

#### 4.2.1 Calcium-associated channel/receptor inhibitors

##### 4.2.1.1 ASICs

ASIC1a inhibitors such as amiloride and its analogs and PcTx1 inhibit glioblastoma proliferation *in vitro* (Escoubas et al., 2000; Hegde et al., 2004). Amiloride can also reduce the rate of metastasis in mice (Matthews et al., 2011). Inhibition of ASIC2 channels by recombinant analogs of Manbalkin-1 can control the carcinogenic process of leukemia (Bychkov M. L. et al., 2020), glioma (Bychkov M. et al., 2020) and melanoma (Bychkov et al., 2021) cells. The specific ASIC1a inhibitor Mambalkin-2 can inhibit the growth and migration of glioma cells and lung adenocarcinoma cells (Bychkov M. et al., 2020; Sudarikova et al., 2022).

##### 4.2.1.2 TRP channels

The TRPC antagonist SKF-96365 has been used to inhibit the proliferation of glioblastoma (GBM) cells in many previous studies, showing antitumor effects (Bomben and Sontheimer, 2008; Chigurupati et al., 2010; Ding et al., 2010; Wu et al., 2021). SKF-96365 is also used as a CRAC channel blocker (Cheng et al., 2013; Jing et al., 2016a). Casazepine (CPZ), a TRPV antagonist, has been shown to effectively inhibit the growth of oral squamous cell carcinoma (OSCC) *in vivo* (Gonzales et al., 2014). Subcutaneous injection of CPZ significantly inhibited the growth of PC-3 tumors (Sánchez et al., 2006). Interestingly, TRP agonists also showed antitumor effects, with the TRPV4 agonist GSK1016790A reducing breast cancer cell viability, and endogenous overexpression of TRPV4 was evident *in vitro*, leading to reduced tumor growth *in vivo*. TRPV4 is able to regulate tumor angiogenesis and vascular maturation, so GSK1016790A has been proposed to be used together with other anticancer drugs, such as cisplatin, to achieve more effective cancer therapy (Peters et al., 2017). In addition, activation of TRPV4 normalizes tumor vasculature, thereby delaying malignant progression (Adapala et al., 2016). However, this activation may also favor cancer cell proliferation, be less sensitive to cell death induction, and favor cancer cell resistance (Wu et al., 2021). The reason for this phenomenon can be explained by the different research methods used to study TRPV4. Another study showed that TRPV4 expression downregulates angiogenesis and that TRPV4 expression is downregulated and Ca^2+^ influx is reduced in tumor endothelial cells compared to healthy endothelial cells. In mouse angiogenesis studies, TRPV4 knockout mice showed an increased number of new vasculature (Adapala et al., 2016). This suggests that basal activation of TRPV4 may inhibit angiogenesis, whereas over-activation of TRPV4 by agonists increases angiogenesis and induces vascular normalization, thereby enhancing the efficacy of cisplatin.

##### 4.2.1.3 VDCC

As mentioned earlier, as increasing evidence has revealed the important role of VGCC in many cancers, many researchers have begun to investigate drugs acting on VGCC for cancer treatment (Kale et al., 2015). A new compound derived from Mibependil, NNC-55-0396, has been developed to selectively target Ca^2+^ channels. This newly derived compound appears to be a promising chemotherapeutic agent due to its ability to efficiently inhibit angiogenesis in cancer cell lines with minimal off-target effects (Kale et al., 2015; Kim et al., 2015).

##### 4.2.1.4 P2X receptors

P2X3 and P2X2/3 are highly expressed in bone cancer pain animals and induce pain, while a P2X3 receptor antagonist (A-317491) can reduce P2X3 and P2X2/3 overexpression and inhibit early bone cancer pain. A recent study found that folic acid alleviated bone cancer pain by downregulating P2X2/3 receptor expression in rats, and continuous folic acid treatment reduced P2X2/3 receptor expression and ameliorated bone cancer pain (Gu et al., 2023). However, the mechanism by which folate downregulates P2X2/3 receptors in bone cancer pain remains unknown. Further studies are needed to address these results as evidence that P2X2/3 receptors contribute to bone cancer pain and suggest that P2X2/3 receptors may be an effective therapeutic target for pain relief in cancer patients. Folic acid treatment has a slow onset of action but a long duration of analgesia, whereas P2X3/2 receptor antagonists have a rapid onset but a short duration of analgesia. These differences suggest that the combination of folic acid and P2X3/2 receptor antagonists may have the best therapeutic effect on bone cancer pain. Brilliant blue G is a P2X7 antagonist that has an inhibitory effect on glioma growth. Emodin is a nonspecific P2X inhibitor that reduces P2X7-mediated cancer cell migration. Many P2X7 receptor modulators, such as antagonists A-438079 and A-740003, are primarily used for pain relief. Since P2X receptors play an important role in a certain number of cancers, it is reasonable to believe that these antagonists may also be potentially effective anticancer agents (Adinolfi et al., 2015).

##### 4.2.1.5 SOCE

In many of targeted CRAC or SOCE known compounds, Orai channel blocker research is the most widespread. In an earlier study, Yang et al. (2009) found that the ORAI1 inhibitor SKF96365 blocked breast cancer cell migration and metastasis. Similar antitumor effects have been reported in colorectal cancer, where SKF96365 induces cell cycle arrest and apoptosis (Jing et al., 2016a). These experiments demonstrated the antitumor effect of blocking CRAC channels. Another commonly used SOCE inhibitor is 2-APB, which was initially identified as a noncompetitive antagonist of IP3R (Maruyama et al., 1997). Although some studies have shown that 2-APB effectively inhibits cancer cell proliferation and tumor progression, its nonselective and multitargeted nature makes it unsuitable for chemotherapy (Sakakura et al., 2003; Wang J. Y. et al., 2015). Two derivatives of 2-APB, i.e., DPB162-AE and DPB163-AE, showed higher potency and specificity in inhibiting SOCE (Guo et al., 2019). DPB162-AE has been shown to have higher SOCE blocking efficacy than 2-APB and strongly affect the proliferation of MDA-MB-231 breast cancer cells (Schild et al., 2020). RO2959 is a novel, potent and selective SOCE inhibitor. RO2959 has been shown to block ORAI1 and STIM2 expression and calcium influx in CHO cells (Chen et al., 2013). However, the *in vivo* efficacy and antitumor effects of RO2959 remain unclear.

##### 4.2.1.6 GPCRs

As mentioned above, proton-sensing GPCRs are a key channel for tumor cells to sense acidity and induce calcium influx in the acidic microenvironment, which suggests that proton-sensing GPCRs have the potential to be a new target for the development of anticancer drugs, and may also be a part of combination therapy. Small molecule modulators of proton-sensing GPCRs are currently under active development and evaluation. Transcriptome analysis revealed that Conophylline (CnP), a vinca alkaloid derived from leaves, strongly inhibited GPR68 in cancer-associated fibroblasts (CAFs) and consequently inhibited the hepatocellular carcinoma (HCC)-promoting effect of CAFs. Combination therapy with CnP and existing anticancer drugs may be a promising strategy for the treatment of refractory HCC associated with activated CAFs (Yamanaka et al., 2021). Recent studies have found that the OGR1 antagonist GPR68-I and GPR4 antagonist Compound 13 can reduce the severity of intestinal inflammation in mouse colitis models, and targeting OGR1 with small molecule inhibitors may be a new therapeutic approach for the treatment of IBD (Sanderlin et al., 2019; de Vallière et al., 2021). The GPR4 antagonist compound 13 also reduced inflammation in arthritis models and angiogenesis in mouse implanted models (Velcicky et al., 2017). Compound 39c, also a GPR4 antagonist, reduced VEGF-induced angiogenesis and alleviated arthritis pain in rats (Miltz et al., 2017). NSC745885, a TDAG8 antagonist, can affect cAMP formation in HEK293T cells and reduce mechanical hyperalgesia and inflammation in mouse joints (Kung et al., 2020). However, to date, few compounds have been identified as ligands/modulators of selective proton-sensing GPCRs. We believe that these compounds may be candidates for anticancer therapy, and therefore, further exploration of effective anticancer drugs targeting proton-sensing GPCRs is needed (Cao et al., 2021).

#### 4.2.2 Drug repurposing

Given that Ca^2+^ signaling is widely involved in the development of tumor cells, some anticancer drugs can also regulate intracellular Ca^2+^ levels. For example, 5-fluorouracil (5FU) is an approved anticancer treatment for multiple cancer types. It has been observed that 5FU mediates HCC cell death by reducing Ca^2+^ influx. Administration of 5FU reduced ORAI1 levels and induced cell death by inhibiting the PI3K/AKT/mTOR pathway (Tang et al., 2017). In contrast, in colon cancer cells, 5FU activates calmodulin by increasing intracellular Ca^2+^ and then triggers apoptosis (Can et al., 2013). Similar effects have been found with the clinical chemotherapy drug cisplatin (Shen et al., 2016). Paclitaxel is widely used to treat ovarian, breast, neck, and head cancers. Taxol was found to induce Ca^2+^ release from the ER via the IP3R pathway (Boehmerle et al., 2006). Several FDA-approved drugs for other diseases (e.g., leflunomide, tolvaptan, and teriflunomide) also affect intracellular calcium levels (Rahman and Rahman, 2017; Frandsen et al., 2020). In addition, many CCBs and other calcium antagonists are also able to inhibit P-gp-mediated drug efflux and are modulators of MDR (Hamilton et al., 1993). For example, the L-type CCB Verapamil can downregulate P-gp expression in A704 and Caki-1 cells, thereby inhibiting MDR in renal cell carcinoma (Yu et al., 2000). Similarly, pretreatment of cancer cells with verapamil effectively reversed MDR to doxorubicin in ovarian cancer (Zheng et al., 2018). Similar effects were observed with nifedipine and diltiazem (Chiu et al., 2010; Yanase et al., 2015; Lee et al., 2020). All this evidence suggests that drug reuse seems to be another possible approach to find effective therapeutic modalities to target calcium imbalance in the acidic tumor microenvironment. New drug development is a difficult and time-consuming process that requires rigorous research, trials, and regulatory processes. Therefore, finding new indications for development from approved drugs may be a powerful and efficient way to develop new drugs.

## 5 Discussion

Empirical evidence from the past few decades supports the central role of Ca^2+^ in human disease, especially cancer. Furthermore, similar to many of the examples outlined above, the acidic microenvironment of tumors promotes tumor initiation and progression by increasing intracellular Ca^2+^ levels through Ca^2+^ channels/receptors. Therefore, the calcium signaling pathway is a potential target for the development of novel anticancer therapies, and as summarized in this review, several agents with therapeutic potential are currently in clinical trials. However, since many Ca^2+^ channels/transporters/pumps may play a role in normal physiology and normal cellular function, targeting these processes is difficult because these channels are expressed in many tissues and Ca^2+^ channel/receptor inhibitors may also cause harm to normal cells. Recent studies provide a possible solution. For example, the combination of channel blockers with advanced drug delivery models, such as nanocavars, can effectively modulate the activity of these channels as well as the associated cellular processes (Huang et al., 2022). Alternatively, the combination of conventional chemotherapy drugs with Ca^2+^ channel blockers can sensitize drug-resistant cancer cells to chemotherapeutic drugs. In addition, although certain calcium channels/signals of cancer cells may be candidate targets, they may be needed for other chemotherapeutic agents. Therefore, drug interactions should be fully considered in clinical practice.

The exploration of calcium signaling mechanisms is still at the Frontier of cancer research, and this research field is still in its infancy. The channels/receptors of calcium influx induced by the acidic tumor microenvironment and their signaling pathways may provide promising clues for new cancer therapies in the coming years. More work is needed to explore and better understand the multiple molecular mechanisms that affect tumor development, maintenance, and metastasis in the hope of discovering more highly effective antitumor agents.
